# Photostabilizing Efficiency of Poly(vinyl chloride) in the Presence of Organotin(IV) Complexes as Photostabilizers

**DOI:** 10.3390/molecules21091151

**Published:** 2016-08-30

**Authors:** Mustafa M. Ali, Gamal A. El-Hiti, Emad Yousif

**Affiliations:** 1 Department of Chemistry, College of Science, Al-Nahrain University, Baghdad 64021, Iraq; mustafa991.m.a@gmail.com; 2 Cornea Research Chair, Department of Optometry, College of Applied Medical Sciences, King Saud University, P.O. Box 10219, Riyadh 11433, Saudi Arabia

**Keywords:** poly(vinyl chloride), photostabilizers, organotin complexes, atomic force microscope, irradiation

## Abstract

Three organotin complexes containing furosemide as a ligand (L), Ph_3_SnL, Me_2_SnL_2_ and Bu_2_SnL_2_, were synthesized and characterized. Octahedral geometry was proposed for the Me_2_SnL_2_ and Bu_2_SnL_2_, while the Ph_3_SnL complex has trigonal bipyramid geometry. The synthesized organotin complexes (0.5% by weight) were used as additives to improve the photostability of poly(vinyl chloride), PVC, (40 μm thickness) upon irradiation. The changes imposed on functional groups, weight loss and viscosity average molecular weight of PVC films were monitored. The experimental results show that the rate of photodegradation was reduced in the presence of the organotin additives. The quantum yield of the chain scission was found to be low (9.8 × 10^−7^) when Ph_3_SnL was used as a PVC photostabilizer compared to controlled PVC (5.18 × 10^−6^). In addition, the atomic force microscope images for the PVC films containing Ph_3_SnL_2_ after irradiation shows a smooth surface compared to the controlled films. The rate of PVC photostabilization was found to be highest for Ph_3_SnL followed by Bu_2_SnL_2_ and Me_2_SnL_2_. It has been suggested that the organotin complexes could act as hydrogen chloride scavengers, ultraviolet absorbers, peroxide decomposers and/or radical scavengers.

## 1. Introduction

Poly(vinyl chloride) (PVC) has unique physical and mechanical properties and is widely utilized as a thermoplastic material [1]. PVC polymeric materials come next to polyolefins in terms of global production and consumption [2]. It has various outdoor applications, mainly in construction materials [3,4,5]. PVC however undergoes photochemical degradation when exposed to sunlight or high temperatures for long periods of time [6]. This photodegradation process can lead to changes in the mechanical and physical properties of PVC [7]. Conjugated double bonds could be formed within PVC chains due to dehydrochlorination [8]. This process commonly takes place due to the presence of impurities and/or defects produced within the polymeric chains during synthesis [9,10]. Cross-linking and a reduction in the average molecular weight also occur within PVC chains due to photodegradation processes [6,7,8]. The poor stability of PVC hinders its use in outdoor applications. It is therefore important to photostabilize PVC to enable its uses in harsh conditions such as high temperatures.

Commercial stabilizers such as plasticizers can be used to enhance photostability of PVC [11]. Photostabilizers including Schiff base complexes [12,13,14], aromatics [15,16,17] and heterocycles [18,19,20] have been used in various studies in order to increase PVC photostability. Other additives include inorganic salts and metal complexes are also common [21,22,23,24,25].

Organotin complexes can be used in a variety of different applications. The diorganotin(IV) additives containing benzamidoacetic acid have been used to stabilize the PVC [26]. In this work, we report the use of di- and triorganotin(IV) complexes containing furosemide as photostabilizers for PVC as part of our general interest in the field of polymeric materials [27,28,29,30,31].

## 2. Results and Discussion

### 2.1. Synthesis

**Scheme 1:**
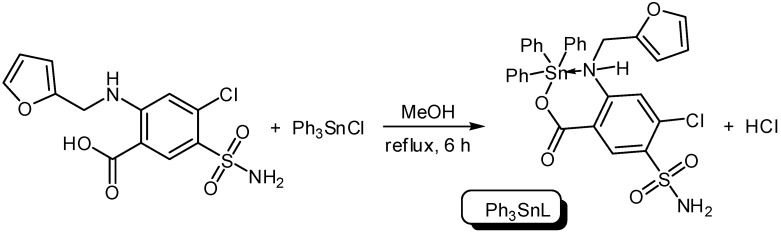
Synthesis of triphenyltin (Ph_3_SnL) complex.

**Scheme 2:**
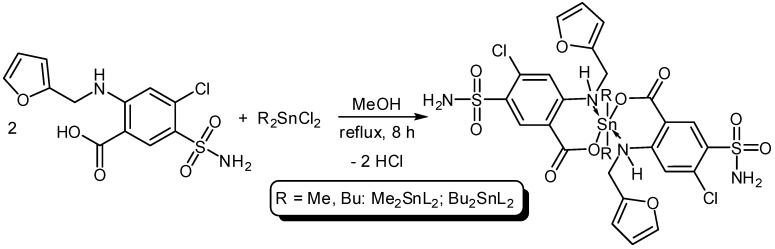
Synthesis of dimethyltin (Me_2_SnL_2_) and dibutyltin (Bu_2_SnL_2_) complexes.

Three organotin complexes, Ph_3_SnL, Me_2_SnL_2_ and Bu_2_SnL_2_, were synthesized from reactions between diorganotin dichlorides or triorganotin chloride with furosemide as a ligand (L) in methanol under reflux conditions for 6–8 h (Scheme 1 and Scheme 2). In the case of Ph_3_SnL, a 1:1 molar ratio of L and Ph_3_SnCl was used, while, a 2:1 molar ratio of L and R_2_SnCl_2_ (R = Me, Bu) was used for the production of Me_2_SnL_2_ and Bu_2_SnL_2_. The structures of Sn(IV) complexes were characterized by elemental analysis and various spectroscopy methods.

### 2.2. Elemental Analysis

The purity of the Sn(IV) complexes was checked by elemental analyses. In addition, flame atomic absorption spectroscopy was used to estimate the tin content within the Sn(IV) complexes. The observed values were generally in agreement with the calculated ones, although it should be noted that the observed carbon content in the Ph_3_SnL complex was significantly less than the calculated one. This could be due to the presence of some impurities and/or solvent. The elemental analyses data (C, H, N, S and Sn %), yields and melting points for Sn(IV) complexes along with those for the ligand are reported in Table 1.

### 2.3. Infrared Spectroscopy

FTIR spectroscopy gives valuable information about the coordination modes within organotin complexes. The FTIR measurements (4000–400 cm^−1^) were performed using the KBr disc method. The FTIR spectroscopic data for L and synthesized organotin(IV) complexes are reported in Table 2.

The IR spectral data for L were compared with those for di- and triorganotin(IV) complexes in order to confirm the binding modes within the synthesized complexes. The IR spectrum of the ligand showed sharp characteristic absorption bands due to asymmetric NH_2_ (3398 cm^−1^) and symmetric NH_2_ (3282 cm^−1^) stretching vibrations. The IR spectra of organotin complexes suggest that the –SO_2_NH_2_ group was not involved in the coordination process with the Sn atom. The peak appearing at 3352 cm^−1^ (secondary NH stretching) for the ligand was shifted slightly in the organotin complexes which is an indication that the NH has coordinated to the Sn(IV) atom. The asymmetric vibrations band for carbonyl group (*ν*_as_ COO) in ligand appeared at 1670 cm^−1^. This peak shifted to higher frequencies for the tin complexes (1672–1679 cm^−1^). The symmetric stretching vibrations band (1562 cm^−1^) for the carboxyl group (*ν*_s_ COO) in the ligand has been shifted to higher frequencies (1566 cm^−1^) in the case of Ph_3_SnL and Bu_2_SnL_2_ and to lower frequency (1558 cm^−1^) in the case of Me_2_SnL_2_. The interaction between the carboxylate oxygen and tin atom can be established from the IR stretching frequencies, Δ*v* (COO) [32]. If Δ*v* [*v*_as_ (COO) − *v*_s_ (COO)] is >350 cm^−1^, the carboxylate group binds in a monodentate mode. The bidentate mode is common when Δ*v* (COO) < 200 cm^−1^. For Δ*v* (COO) less than 200 and more than 350 cm^−1^ an intermediate state between bidentate and monodentate takes place. The Δ*v* (COO) values in the bridging mode have been reported to be more than those in the chelating mode [33].

Three new absorption bands appeared in the IR spectra of the organotin complexes. These bands are attributed to *v* (Sn–N), *v* (Sn–O) and *v* (Sn–C) that resonate within the 408–416, 447–426 and 516–540 cm^−1^ regions, respectively. The appearance of such peaks provides further evidence for the coordination between ligand and Sn(IV). The coordination thus takes place between the tin atom and a secondary amine, carboxylate and alkyl or phenyl groups [34,35].

### 2.4. Ultraviolet-Visible Spectroscopy and Conductivity

The absorption spectra (200–600 nm) for the ligand and its organotin complexes were recorded in DMF (Table 3). The electronic spectrum of ligand shows two absorption bands. The first resonates at 274 nm due to π–π* electronic transition of the aromatic moieties. This band was slightly shifted to higher wavelength after complexation. The second band resonates at 341 nm due to n–π* electronic transition for the carbonyl oxygen non-bonding electrons and has been shifted slightly to higher wavelength within organotin complexes. The molar conductivity for organotin complexes (10^−3^ M) were measured in ethanol at room temperature. The conductivity for Sn(IV) complexes was found to be in the range of 2.1–4.4 μS/cm (Table 3). The organotin complexes were found to be non-electrolyte.

### 2.5. ^1^H-NMR Spectroscopy

The ^1^H-NMR spectra L and its Sn(IV) complexes were recorded in DMSO-*d*_6_ (Table 4) and were consistent with their chemical structures. The changes in the chemical shift values between the ligand and its tin complexes are largely dependent on the environment and can be used as evidence to show that complexation has taken place [36]. The ^1^H-NMR spectrum of the ligand showed an exchangeable singlet which resonates at 13.35 ppm due to the carboxylate proton. Such a signal was absent in the ^1^H-NMR spectra of the organotin complexes. The aromatic protons in Sn(IV) complexes was slightly shifted up-field compared to the corresponding ones in the ligand [37]. In contrast, the NH signal was shifted down-field upon chelation of ligand with Sn(IV) atom. This could be a result of a charge transfer towards the carboxylate group that binds to the highly electropositive Sn atom [35]. The ^1^H-NMR spectra of organotin complexes showed two singlet aromatic protons that resonated within the 7.06–7.10 and 8.38–8.44 ppm regions which were attributed to H-3 and H-6, respectively. They also showed an apparent triplet (*J* = 3.2 Hz) that resonated at 6.38–6.42 ppm which is characteristic for H-4 of a furan. The H-3 of the furan moiety resonated a doublet (*J* = 3.2 Hz) within the 6.31–6.37 ppm region. Moreover, the CH_2_ protons resonated as a doublet (*J* = ca. 6 Hz) at 4.44–4.58 ppm.

### 2.6. Evaluation of Stabilizing Efficiency of PVC by Weight Loss

**Figure 1:**
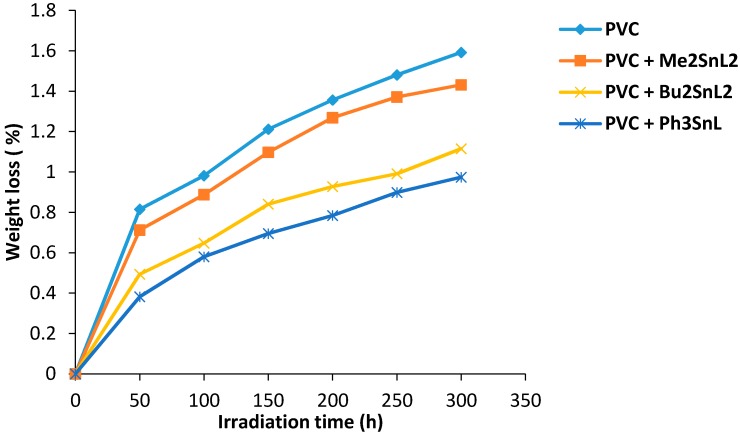
Changes in weight loss of PVC films versus irradiation time.

Photodegradation of PVC is associated with the evaluation of HCl gas (dehydrochlorination) and leads to weight loss. Therefore, the weight loss percentage can be used to measure the degree of PVC photodegradation. It has been reported that additives with a concentration of 0.5% by weight showed the most efficient photostabilization effect when added to PVC [20]. Therefore, di- and triorganotin(IV) additives (0.5% by weight) were added to PVC to produce the films. Their photostabilization effect was investigated for PVC films (40 μm thickness) on irradiation for up to 300 h. The effect of irradiation time (300 h) on weight loss of PVC films (40 μm thickness) is shown in Figure 1. Evidently, the PVC weight loss percentage was lower in the presence of organotin complexes when compared to the blank PVC film. The Ph_3_SnL complex shows the most efficient stabilizing effect compared to the other additives. The Ph_3_SnL complex is believed to be a better radical scavenger compared to other complexes since the extra phenyl ring increases the photostabilization of PVC films on irradiation *via* resonance. The PVC photostabilization efficiency of organotin(IV) complexes was found to follow the order Ph_3_SnL > Bu_2_SnL_2_ > Me_2_SnL_2_.

### 2.7. Evaluation of Stabilizing Efficiency of PVC by FTIR Spectroscopy

**Figure 2:**
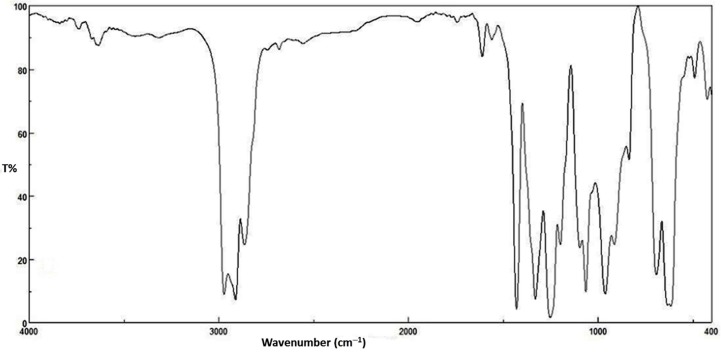
FTIR spectrum of PVC film containing Bu_2_SnL_2_ complex before irradiation.

**Figure 3:**
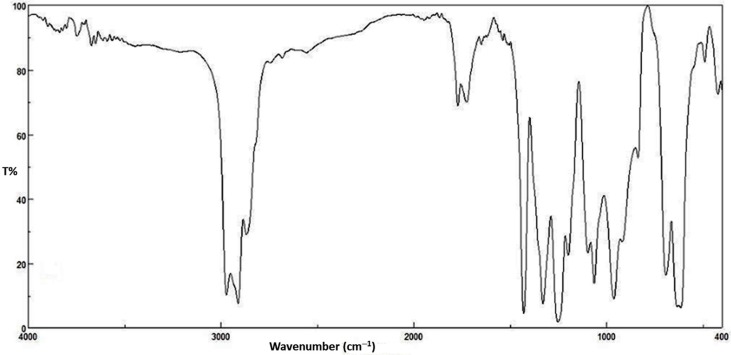
FTIR spectrum of PVC film containing Bu_2_SnL_2_ complex after irradiation.

The photochemical activity of organotin complexes as additives for PVC films photostabilization was investigated using FTIR spectroscopy. The PVC films were irradiated with UV light (λ_max_ = 365 nm) for 300 h. The irradiation of PVC leads to the formation of various functional groups. The IR spectrum of irradiated PVC shows three absorption bands due to formation of carbonyl, polyene and hydroxyl groups. These bands resonate at 1772 (C=O), 1604 (C=C) and 3500 cm^−1^ (OH), respectively [38]. The growth rate of such peaks related to a reference peak (1328 cm^−1^) could be considered as a measure for the photodegrading of PVC. The FTIR spectra of PVC films containing Bu_2_SnL_2_ complex before and after irradiation are represented in Figure 2 and Figure 3, respectively.

**Figure 4:**
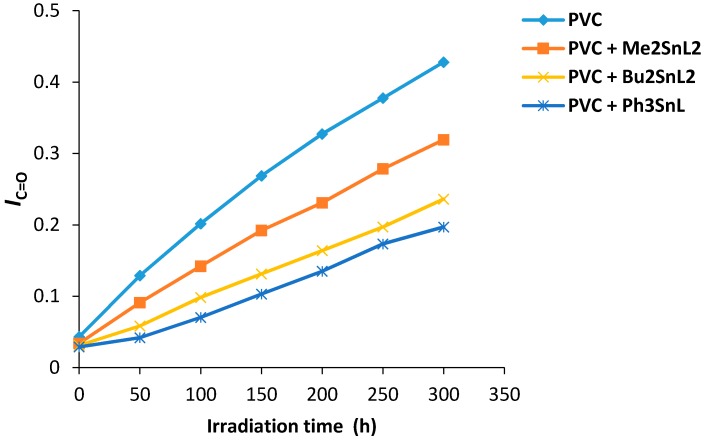
Changes in the *I*_C=O_ index for PVC films versus irradiation time.

The changes in carbonyl group intensity (1722 cm^−1^) for PVC films containing organotin(IV) was monitored on irradiation. The carbonyl index (*I*_C+O_) was calculated and plotted against irradiation time. The growth rate of carbonyl group was lower when Ph_3_SnL, Me_2_SnL_2_ and Bu_2_SnL_2_ (0.5 wt %) were used as additives compared to PVC without additives (Figure 4). The Ph_3_SnL complex was the most efficient additive amongst the ones used in photostabilization of PVC followed by Bu_2_SnL_2_ and Me_2_SnL_2_.

**Figure 5:**
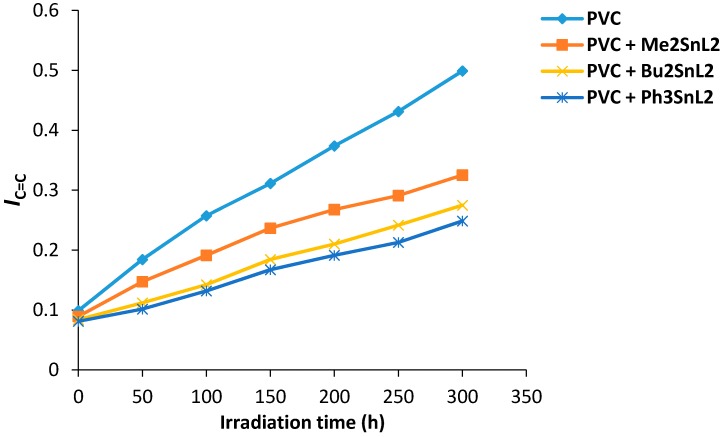
Changes in the *I*_C=C_ index for PVC films versus irradiation time.

Similarly, the changes in the C=C group intensity was monitored and the polyene index (*I*_C=C_) was calculated. Figure 5 shows the changes in *I*_C=C_ of PVC films upon irradiation. All the additives used show stabilization for PVC on irradiation in which triphenyltin(IV) complex was the most effective additive against photodegradation of PVC.

**Figure 6:**
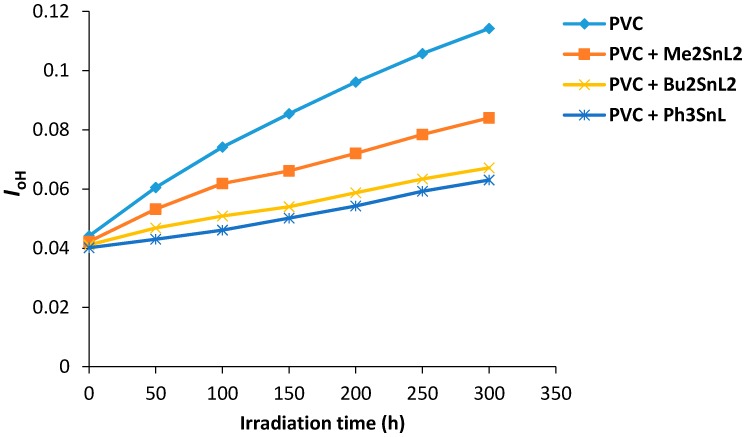
Changes in the *I*_OH_ index for PVC films versus irradiation time.

Figure 6 shows the changes in hydroxyl index (*I*_OH_) of PVC films containing organotin(IV) complexes during irradiation. Again the additives used, Ph_3_SnL, Me_2_SnL_2_ and Bu_2_SnL_2_, showed lower *I*_OH_ growth compared to PVC (control).

### 2.8. Evaluation of Stabilizing Efficiency of PVC by Variation in Molecular Weight

**Figure 7:**
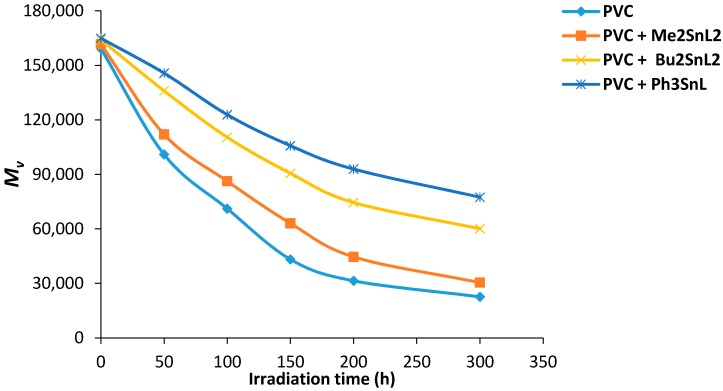
Changes in ${\bar{M}}_{V}$ for PVC films versus irradiation time.

Viscosity average molecular weight (${\bar{M}}_{V}$) can be measured from intrinsic viscosity using Equation (5). The changes in ${\bar{M}}_{V}$ for PVC films (40 μm thickness) containing organotin complexes (0.5 wt %) was plotted against irradiation time (Figure 7) with a light intensity of 1.052 × 10^−8^ ein·dm^−2^·s^−1^ (THF, 25 °C).

It was noted that some insoluble PVC traces in THF was produced during photolysis process. It was suggested that these residues could possibly be an indication for PVC crosslinking or branching that occurs on irradiation [39]. To clarify this possibility, the number of average chain scission (*S*) [40] was calculated using Equation (1). The *S* value is highly dependent on the viscosity average molecular weight at start (${\bar{M}}_{V,O}$) and at *t* irradiation time (${\bar{M}}_{V,t}$).

(1) $$S={\bar{M}}_{V,O}/{\bar{M}}_{V,t}\;-1$$

**Figure 8:**
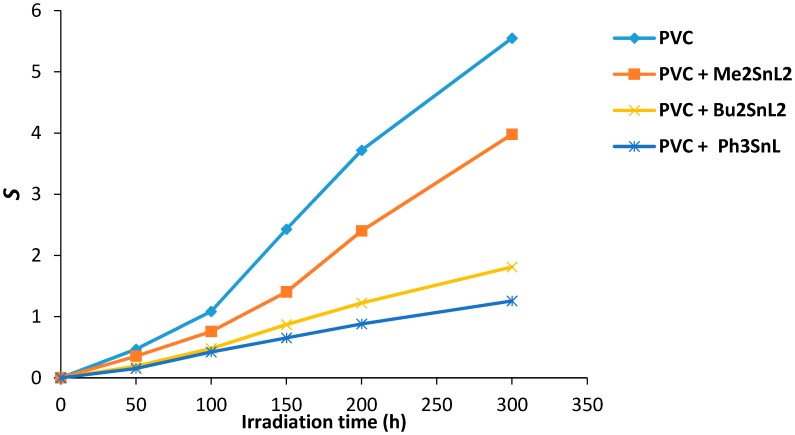
Changes in the main chain scission (*S*) for PVC films versus irradiation time.

Figure 8 shows the effect of irradiation time on the value of S for PVC films containing organotin(IV) complexes. The PVC (control) on irradiation shows a higher degree of branching and/or cross-linking compared to the ones containing additives. The increases in *S* value was sharp for control PVC between 100 and 300 h. Less insoluble residual polymers were observed in PVC containing complexes and in particular those that contain Ph_3_SnL.

The degree of deterioration (α) for PVC films can be calculated using Equation (2). It is dependent on the initial molecular weight (*m*), *S* and ${\bar{M}}_{V}$. The value of α is proportional to the break of randomly distributed weak bond which is fast in the initial stage of irradiation.

(2) $$\alpha =m\times S/{\bar{M}}_{V}$$

**Figure 9:**
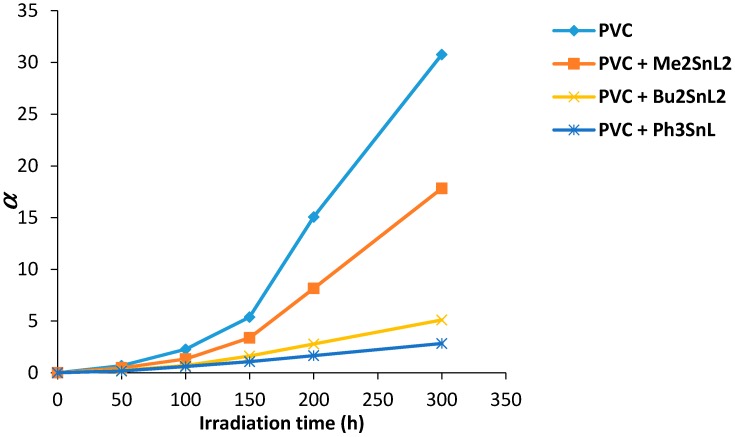
Changes in the degree of deterioration (α) for PVC films versus irradiation time.

The effect of irradiation on PVC films is shown in Figure 9. The change in α value was increased with irradiation time. The increase in α value for PVC (control) was steep at 150 °C and reached a maximum after 300 h. It seems likely that random bond breakage is occurring within PVC chain. The α value was lower for PVC containing Sn(IV) complexes and was minimum in the case of triphenyltin(IV) complex. It is clear that the use of such additives has reduced the photodegradation of PVC films upon irradiation.

The quantum yield of chain scission (Φ_cs_) for PVC films was measured using Equation (6) and represented in Table 5. The Φ_cs_ for PVC (control) was found to be higher (5.18 × 10^−6^) compared to the ones containing Sn(IV) complexes (1.58 × 10^−6^–9.8 × 10^−7^). The higher the Φ_cs_ value the greater the photodegrading of PVC films [13]. The PVC film which contains Ph_3_SnL complex shows the lowest Φ_cs_ value. It can therefore be suggested that this additive inhibits the photodegrading of PVC upon irradiation. The quantum yield (Φ_cs_) increases with increasing temperature [13]. It reaches peak around the melting temperature and glass transition temperature (Tg; 80 °C) of the crystalline and amorphous polymers, respectively. The PVC photolysis was carried out at 35–45 °C which is lower than the *T*g of PVC. Therefore, the dependence of Φ_cs_ on temperature is not significant [41].

### 2.9. Surface Analysis

**Figure 10:**
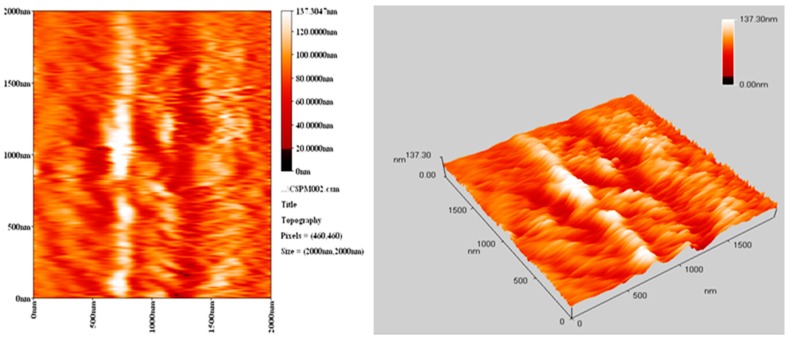
AFM images for PVC film (control) after irradiation (300 h).

**Figure 11:**
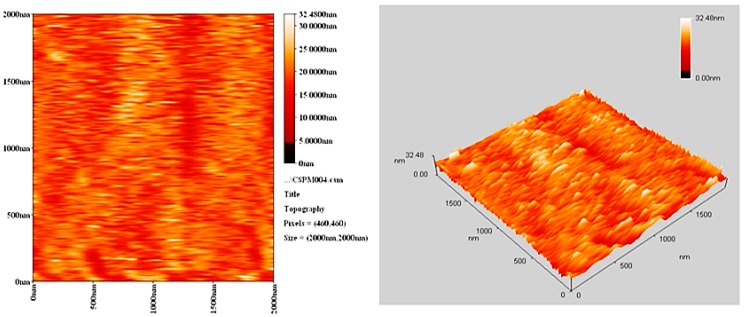
AFM images for PVC film containing Ph_3_SnL complex after irradiation (300 h).

The morphology of PVC surface (area = 5.0 × 5.0 μm^2^) was inspected using an atomic force microscope (AFM). It provides two- and three-dimensional topographic images of the PVC surface on irradiation (300 h). AFM is helpful to measure the pores size and roughness factor (*R*q) of the PVC film [42]. The AFM images for PVC films (control; 40 μm thickness) and that contains Ph_3_SnL (0.5 wt %) as a photostabilizer after irradiation (300 h) are given in Figure 10 and Figure 11, respectively.

The surface of PVC film containing Ph_3_SnL after irradiation was very smooth (*R*q = 2.61). In contrast, the blank PVC film after irradiation has a rough surface (*R*q = 17.3). UV irradiation may have led to bond breakage within polymeric chains and hence the removal of leachable constituents from the PVC surface which can result in a roughened surface [43]. Irradiation of PVC in the absence of photostabilizers could lead to dehydrochlorination which is common at 150 °C and above [44]. It seems likely that the Sn(IV) complexes inhibits the dehydrochlorination process.

### 2.10. Suggested Mechanisms of Photostabilization of PVC

**Scheme 3:**
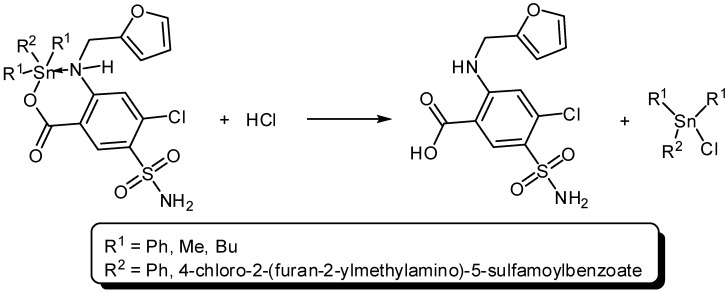
Organotin complexes act as HCl scavengers.

The di- and triorganotin(IV) complexes act as photostabilizers for PVC films and their efficiencies follow the order Ph_3_SnL > Bu_2_SnL_2_ > Me_2_SnL_2_. The three Sn(IV) utilized all reduced PVC photodegradation. Such additives can stabilize the polymeric films based on various mechanisms. Tin is a strong Lewis acid which is also known as a secondary stabilizer and therefore acts as HCl scavenger (Scheme 3). The photostabilization effect could be due to displacement of chlorine atom within PVC chains by carboxylate oxygen. Various organic compounds are known as long-term PVC stabilizers [21].

**Scheme 4:**
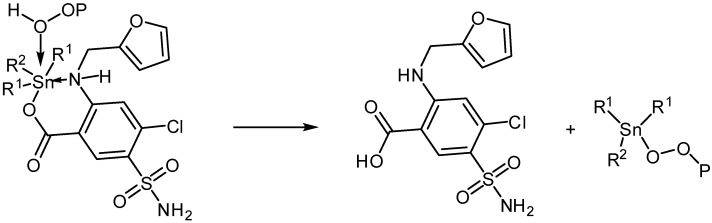
Organotin complexes as peroxide decomposers.

Photooxidation of PVC can take place in the presence of hydroperoxides. Various additives are known for their reactions with hydroperoxides and therefore inhibit photodegradation of polymers [45]. The organotin(IV) complexes are expected to decompose peroxides (Scheme 4) and therefore, stabilize PVC polymeric chains.

**Scheme 5:**
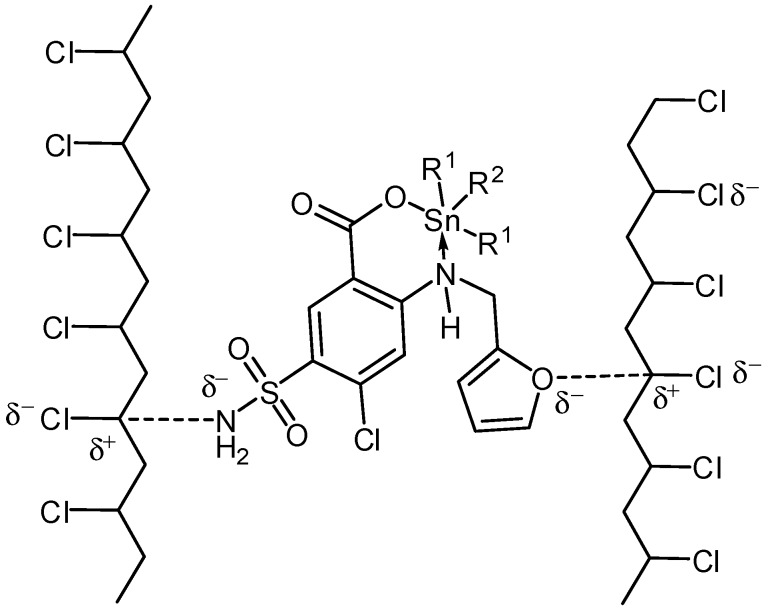
Organotin complexes as primary stabilizers.

Attraction between polarized C–O bonds in furan moiety and S–NH_2_ group within Sn(IV) complexes and polarized C–Cl within PVC chains could stabilize the polymer (Scheme 5). This attraction can aid the conversion of the excited state PVC energy to a level of energy that does not harm the polymeric chain [20].

**Scheme 6:**
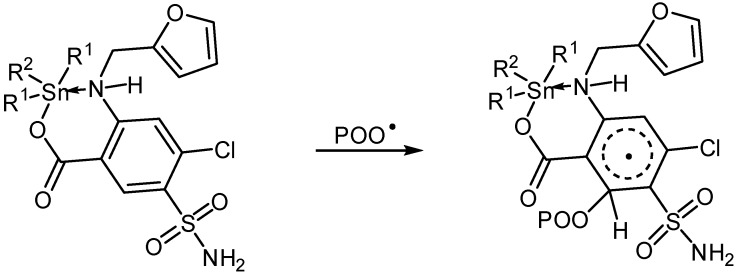
Organotin complexes as radical scavengers.

Sn(IV) complexes could also act as radical scavengers (Scheme 6). The additives could form a complex with a chromophore (POO^•^) in the excited state [46]. This complex could be stabilized via resonance of aryl and furan moieties (Scheme 6).

**Scheme 7:**
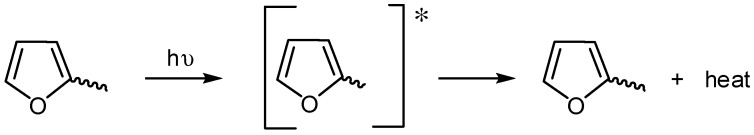
Furan moiety acts as a UV absorber.

Electron rich aromatic moieties within the PVC backbone can absorb UV radiation [20]. These additives can convert the absorbed UV radiation energy into heat energy that is not harmful to PVC. Therefore, it is believed that both furan and aryl ring within Sn(VI) complexes act as UV absorbers (Scheme 7).

## 3. Experimental

### 3.1. Materials

Furosemide, reagents and solvents were purchased from Sigma-Aldrich Chemical Company (Gillingham, UK) and used as received. PVC (*K* value = 67, degree of polymerization = 800) was supplied from Petkim Petrokimya (Istanbul, Turkey).

### 3.2. Synthesis of Triphenyltin(IV) Complex

To a stirred solution of furosemide (0.33 g, 1.0 mmol) in methanol (15 mL) a solution of chlorotriphenylstannane (0.39 g, 1.0 mmol) in methanol (10 mL) was added in a dropwise manner. The mixture was stirred at room temperature for 10 min and then heated under reflux for 6 h. The mixture was filtered and the solvent was removed under reduced pressure. The solid obtained was purified by crystallization using methanol to give white powder of triphenyltin(IV) complex.

### 3.3. Synthesis of Diorganotin(IV) Complexes

To a stirred solution of furosemide (0.66 g, 2.0 mmol) in methanol (20 mL) a solution of dichlorodimethylstannane or dibutyldichlorostannane (1.0 mmol) in methanol (10 mL) was added in a dropwise manner. The mixture was heated under reflux for 8 h. The mixture was filtered and the solvent was removed under reduced pressure. The solid obtained was purified by crystallization using methanol to give the organotin(IV) complexes as off white powders.

### 3.4. Films Preparation

A solution of PVC in tetrahydrofuran (5 g/100 mL) was re-precipitated using ethanol. The PVC was dried under reduced pressure at room temperature for 24 h. PVC films (40 µm thickness) was prepared. The thickness was measured using a Digital Caliper Vernier (Kevelaer, Germany). The PVC films containing organotin complexes (0.5% by weight) were prepared by the evaporation technique at room temperature for 24 h. The residual tetrahydrofuran was removed and the PVC films containing additives were fixed using aluminum plate stands supplied by Q-Panel Company (Homestead, FL, USA) [12].

### 3.5. Accelerated Testing Technique

Accelerated weather-meter QUV tester (Philips, Saarbrücken, Germany) was used to irradiate PVC films. The weather-meter is equipped with two UV-B 313 lamps (290–360 nm) with λ_max_ of 313 nm [13].

### 3.6. Measuring the PVC Photodegradation Rate by FTIR Spectrophotometry

The progress of PVC photodegradation due to irradiation was investigated using a FTIR 8300 Spectrophotometer (Shimadzu, Tokyo, Japan, 4000–400 cm^−1^). The changes in carbonyl (1722 cm^−1^), polyene (1602 cm^−1^) and hydroxyl (3500 cm^−1^) absorption peaks intensity were recorded [47]. The carbonyl (*I*_CO_), polyene (*I*_PO_) and hydroxyl (*I*_OH_) indices were calculated by comparison of the FTIR absorption peaks with the reference peak that resonates at 1328 cm^−1^. The functional group index (*Is*) is the functional group peak absorbance (*As*) divided by the reference peak absorbance (*Ar*). *Is* can be calculated using the baseline method [47] as shown in Equation (3):

(3) Is=As/Ar

### 3.7. Measuring the Photodegradation by Weight Loss

The PVC film weight loss percentage was calculated using Equation (4). It can be calculated from PVC weight before irradiation (*W*_1_) and that after irradiation (*W*_2_) [48].

(4) $$\text{Weight loss }\%\;=\;\left[\left(W_{1}-W_{2}\right)/W_{1}\right]\;\times \;100$$

### 3.8. Measuring the Photodegradation by Morphology Study

The morphology of PVC surface was inspected before and after irradiation using atomic force microscopy (AFM; Veeco, (Plainview, NY, USA)) and a microscope (Meiji Techno, Tokyo, Japan).

### 3.9. Determination of the Average Molecular Weight (${\bar{M}}_{V}$) by the Viscometry Method

The intrinsic viscosity, [η], was calculated using Equation (5).

(5) $$\left[\eta \right]=4.17\;\times \;10^{-4}\;{{\bar{M}}_{V}}^{0.6}$$

The quantum yield of main chain scission (Φ_cs_) [49] is dependent on intrinsic viscosity of PVC before irradiation ($\eta_{o}$), irradiation time (*t*), incident intensity (*I_o_*), ${\bar{M}}_{V,O}$, concentration (*C*) and Avogadro’s number (*A*). The Φ_cs_ was calculated using Equation (6):

(6) ΦCS=(CA/M¯V,O)⌊([ηo]/[η])1α−1⌋/Iot

## 4. Conclusions

Three organotin complexes (Ph_3_SnL, Me_2_SnL_2_ and Bu_2_SnL_2_) were synthesized and characterized. The complexes have been used as additives to stabilize PVC films upon irradiation. The PVC photodegradation rate has been reduced in the presence of organotin complexes (0.5% by weight) upon UV irradiation for 300 h. In addition, the AFM images show a smooth surface for the PVC films containing Ph_3_SnL complex. The most photostable PVC film was the one that contains Ph_3_SnL followed by Bu_2_SnL_2_ and Me_2_SnL_2_. A number of mechanisms was proposed to explain the PVC photostabilization in the presence of Sn(IV) complexes. The additives could act as HCl scavengers, UV absorbers, peroxide decomposers and/or radical scavengers.

## Figures and Tables

**Table 1:** Melting points, yields and elemental analyses for L and its Sn(IV) complexes.

Compound	Mp (°C)	Yield (%)	Elemental Analysis (%) Calculated (Found)				
			C	H	N	S	Sn
L	205–207	—	43.58	3.35	8.47	9.69	—
			(43.19)	(3.34)	(7.93)	(9.44)	
Ph_3_SnL	159–161	73.2	53.09	3.56	4.13	4.72	17.49
			(48.12)	(3.41)	(4.53)	(4.87)	(17.21)
Me_2_SnL_2_	184–186	67.4	38.64	3.47	6.93	7.93	14.69
			(39.94)	(3.27)	(6.31)	(8.92)	(14.77)
Bu_2_SnL_2_	194–197	80.7	43.07	4.29	6.28	7.19	13.28
			(43.32)	(3.44)	(7.91)	(7.94)	(12.28)

**Table 2:** FTIR spectroscopic data for L and its organotin(IV) complexes.

Vibrational Mode	Compound (Wavenumber; cm^−1^)			
	L	Ph_3_SnL	Me_2_SnL_2_	Bu_2_SnL_2_
*v* (NH)	3352	3350	3344	3348
*v*_as_ (COO)	1670	1674	1672	1679
*v*_s_ (COO)	1562	1566	1558	1566
Δ*v* (COO) = *v*_as_ (COO) − *v*_s_ (COO)	108	108	114	113
*v* (Sn–C)	—	540	536	516
*v* (Sn–O)	—	426	432	447
*v* (Sn–N)	—	416	408	408

**Table 3:** Electronic spectral data and conductivity for L and its organotin(IV) complexes.

Compound	Absorption nm (n–π* and π–π*; cm^−1^)	Conductivity (μS/cm)
L	341, 274	—
Ph_3_SnL	348, 277	2.1
Me_2_SnL_2_	342, 376	4.4
Bu_2_SnL_2_	344, 275	2.4

**Table 4:** ^1^H-NMR spectral data for ligand and organotin complexes.

Compound	^1^H-NMR (300 MHz: DMSO-*d*_6_, δ, ppm, *J* in Hz)
L	13.35 (s, exch., CO_2_H), 8.51 (t, *J* = 6.0 Hz, exch., NH), 8.39 (s, H-6), 7.62 (d, *J* = 3.2 Hz, H-5 of furan), 7.33 (s, exch., NH_2_), 7.05 (s, H-3), 6.40 (app. t, *J* = 3.2 Hz, H-4 of furan), 6.36 (d, *J* = 3.2 Hz, H-3 of furan), 4.64 (d, *J* = 6.0 Hz, CH_2_)
Ph_3_SnL	8.61 (t, *J* = 6.2 Hz, exch., NH), 8.41 (s, H-6), 7.82–7.33 (m, 3 Ph and H-5 of furan), 7.22 (s, exch., NH_2_), 7.10 (s, H-3), 6.38 (app. t, *J* = 3.2 Hz, H-4 of furan), 6.31 (d, *J* = 3.2 Hz, H-3 of furan), 4.44 (d, *J* = 6.2 Hz, CH_2_)
Me_2_SnL_2_	8.61 (t, *J* = 6.1 Hz, exch., NH), 8.44 (s, H-6), 7.62 (d, *J* = 3.2 Hz, H-5 of furan), 7.34 (s, exch., NH_2_), 7.06 (s, H-3), 6.42 (app. t, *J* = 3.2 Hz, H-4 of furan), 6.35 (d, *J* = 3.2 Hz, H-3 of furan), 4.58 (d, *J* = 6.1 Hz, CH_2_), 1.23 (s, Me)
Bu_2_SnL_2_	8.64 (t, *J* = 6.0 Hz, exch., NH), 8.38 (s, H-6), 7.61 (d, *J* = 3.2 Hz, H-5 of furan), 7.29 (s, exch., NH_2_), 7.06 (s, H-3), 6.41 (app. t, *J* = 3.2 Hz, H-4 of furan), 6.37 (d, *J* = 3.2 Hz, H-3 of furan), 4.48 (d, *J* = 6.0 Hz, CH_2_), 1.55 (t, *J* = 7.2 Hz, CH_2_), 1.37–1.31 (m, C*H*_2_C*H*_2_Me), 0.85 (t, *J* = 7.2 Hz, Me)

**Table 5:** Quantum yield (Φ_cs_) for the chain scission for PVC film (40 µm) containing metal complexes (0.5 wt %) after irradiation (300 h).

Compound	Quantum Yield of Main Chain Scission (Φ_cs_)
PVC (control)	5.18 × 10^−6^
PVC + Ph_3_SnL	9.80 × 10^−7^
PVC + Me_2_SnL_2_	3.79 × 10^−6^
PVC + Bu_2_SnL_2_	1.58 × 10^−6^
